# Bridging the Gap: A Cross-Sectional Study of Hemodialysis Services and Patient Psychosocial Burden in Semi-urban Punjab, Pakistan

**DOI:** 10.7759/cureus.108166

**Published:** 2026-05-03

**Authors:** Muhammad Arslan Shahid, Zeerak Laila, Muhammad Zeeshan Arif, Yousuf Shahjahan

**Affiliations:** 1 Department of Surgery, THQ Hospital, Chunian, PAK; 2 Department of Anesthesiology, St. Vincent's University Hospital, Dublin, IRL; 3 Faculty of Medicine, Ala-Too International University, Bishkek, KGZ

**Keywords:** arteriovenous fistula, clinical audit, dialysis adequacy, end-stage renal disease, hemodialysis, infection control, resource-limited settings, rural pakistan

## Abstract

Background

Chronic kidney disease progressing to end-stage renal disease (ESRD) poses a significant public health challenge, particularly in resource-constrained settings. This clinical audit aimed to evaluate patient satisfaction, clinical practices, and the psychosocial burden among hemodialysis patients at secondary care facilities in Pakistan, benchmarking against Kidney Disease Outcomes Quality Initiative (KDOQI) and Kidney Disease: Improving Global Outcomes (KDIGO) standards.

Methods

A cross-sectional clinical audit was conducted among 100 ESRD patients receiving maintenance hemodialysis. Data regarding demographic profiles, clinical care standards, patient education, and psychosocial experiences were collected using a validated, interviewer-administered questionnaire. Patient satisfaction was assessed using a standardized five-point Likert scale.

Results

The cohort (mean age: 42.4 ± 8.2 years; 65.0% male) demonstrated high satisfaction with technical care. Arteriovenous fistulas were utilized by 100% (n = 100) of patients. Maximum satisfaction scores (5/5) were reported by 90% (n = 90) for cannulation technique and overall care. Safety protocol adherence was strict, with 100% confirming the use of dedicated machines for hepatitis B/hepatitis C-positive cases. However, a severe psychosocial and economic burden was observed: 100% of the cohort reported current unemployment, and 100% reported experiencing severe treatment-related stress, anxiety, or depression.

Conclusions

Secondary care hemodialysis centers in this cohort demonstrated high technical proficiency and strict adherence to biomedical safety protocols. However, this clinical excellence contrasts with universal patient psychosocial distress and economic vulnerability, highlighting an urgent need to integrate routine mental health and socioeconomic support into the standard renal care model.

## Introduction

Chronic kidney disease (CKD) has emerged as a major global public health priority, characterized by the progressive loss of renal function and high morbidity and mortality rates [1]. When CKD advances to end-stage renal disease (ESRD), life-sustaining renal replacement therapy, most commonly in the form of maintenance hemodialysis, becomes a clinical necessity for survival [2]. Beyond its primary role in solute clearance and fluid homeostasis, dialysis significantly impacts a patient’s systemic health, influencing cardiovascular stability, mineral bone metabolism, and overall nutritional status. However, the complexity of dialysis care extends far beyond the clinical procedure, encompassing rigorous infection control and strict adherence to pharmacological and dietary regimens [3].

Over the past two decades, the global prevalence of ESRD has surged, driven by the rising incidence of diabetes mellitus and hypertension, the leading etiologies of renal failure worldwide [4]. This “growing epidemic” of kidney failure poses a disproportionate challenge to low- and middle-income countries (LMICs), where the high cost of treatment and limited healthcare infrastructure often result in suboptimal patient outcomes [5,6]. In these regions, the quality of dialysis care, both technical and psychosocial, is a critical determinant of long-term survival.

Pakistan experiences one of the highest burdens of ESRD in South Asia, with an estimated annual incidence that continues to climb due to the uncontrolled prevalence of metabolic disorders [7]. Despite the proliferation of private and public dialysis centers, patients in Pakistan face unique challenges. While international standards demand rigorous safety protocols, local resource constraints often lead to inconsistencies in adherence to infection control guidelines, alongside high out-of-pocket expenditures and a significant lack of integrated psychosocial support. Furthermore, factors such as limited health literacy regarding medication side effects, dietary fluid restrictions, and technical complications of vascular access, such as cannulation-related trauma, further complicate the patient experience [8].

Addressing these multifaceted challenges requires a comprehensive evaluation of patient-centered metrics. While clinical markers are vital, they are insufficient to gauge the holistic success of a dialysis program; a deep understanding of the patient’s socioeconomic and psychological experience is essential for improving treatment compliance and quality of life [9,10]. This study seeks to evaluate the intersection of clinical standards and patient psychosocial burden within secondary care facilities in the Punjab province, providing a baseline for future quality improvement.

## Materials and methods

Study design and population

This study was designed as a descriptive cross-sectional study utilizing an audit-based benchmarking approach. The rationale was to evaluate the quality of hemodialysis services at secondary care facilities in semi-urban Punjab (Kasur District), Pakistan, by benchmarking local practices against international Kidney Disease Outcomes Quality Initiative (KDOQI) and Kidney Disease: Improving Global Outcomes (KDIGO) standards. Data collection was conducted over a period of two months, from December 1, 2025, to February 1, 2026. The target population included patients diagnosed with ESRD receiving maintenance hemodialysis at these centers.

Sample size and sampling technique

A convenience sampling technique was employed, enrolling 100 consecutive patients who met the inclusion criteria during the study period. This sample represented a convenience census of all eligible and consenting patients receiving maintenance hemodialysis at the participating facilities during the two-month study window.

Selection of participants

Participants were selected based on specific eligibility criteria to ensure a homogenous study population. The inclusion criteria comprised patients aged 18 years or older diagnosed with ESRD and receiving maintenance hemodialysis for a minimum of three months. Only patients willing to provide informed consent were enrolled. The exclusion criteria included patients presenting with acute kidney injury requiring only temporary dialysis support and individuals with severe cognitive impairment or documented psychiatric disorders that would preclude accurate completion of the study questionnaire.

The 100% arteriovenous fistula (AVF) utilization rate observed is a reflection of the local referral pathway; at these secondary care facilities, maintenance hemodialysis is primarily reserved for stable patients with mature vascular access, while patients requiring acute catheter-based initiation are typically referred to larger tertiary centers.

Data collection tool

Data were collected using a structured, interviewer-administered questionnaire designed specifically for this study. The questionnaire was developed after reviewing relevant literature and international dialysis guidelines. Content validity was established through expert review by the clinical team. To ensure linguistic and cultural appropriateness, the questionnaire was translated into Urdu and Punjabi and back-translated to verify semantic accuracy. Prior to formal data collection, a pilot test was conducted on a small subset of patients (n = 5, excluded from final analysis) to assess comprehensibility, leading to minor phrasing adjustments for clarity. While the questionnaire was modeled after international clinical standards, it functioned as a structured screening survey designed to capture perceived patient burden rather than a clinical diagnostic tool for psychiatric disorders.

Questionnaire structure

The questionnaire was structured into four thematic sections. The socioeconomic and demographic profile section included age, gender, marital status, employment, and monthly income (PKR). The technical clinical care section included dialysis frequency, primary vascular access type, and adherence to unit-specific infection control protocols. The patient experience and education section was evaluated using a standardized five-point Likert scale to capture nuance in physician explanations, staff behavior, and education regarding self-care. The psychosocial factors section captured self-reported perceived stress, anxiety, or depression using Likert-scale responses to identify generalized psychological burden (Appendix A).

Data analysis

Data were entered into Microsoft Excel (Microsoft Corporation, Redmond, WA, USA) and analyzed using IBM SPSS Statistics for Windows, version 27.0 (released 2019; IBM Corp., Armonk, NY, USA). Descriptive statistics were applied; categorical variables were reported as frequencies and percentages, while continuous variables (e.g., age) were summarized using mean and SD. Patient satisfaction was assessed using a standardized five-point Likert scale (1 = very dissatisfied, 5 = very satisfied). To assess the likelihood of facility recommendation, a 10-point scale was utilized (0 = not at all likely, 10 = extremely likely). Comorbidity data were categorized into mutually exclusive groups to account for overlapping clinical profiles.

Data handling and quality control

Data were initially recorded on hard-copy forms and subsequently entered into a secure, password-protected electronic database. Entry was cross-checked by a second independent researcher. Face-to-face interviews virtually eliminated missing data; the format resulted in a 100% completion rate for all primary variables.

Mitigation of interviewer and response bias

To mitigate interviewer bias, data collection followed a highly standardized script with neutral, non-leading tones. To minimize response bias (social desirability), all interviews were conducted in a private setting independent of the patient’s primary treating physicians and nursing staff. Participants were explicitly counseled that their responses were confidential, anonymized, and would have no punitive impact on their clinical care.

Ethical approval

Ethical approval was granted by the institutional administration and ethical review committees of THQ Hospital Chunian and DHQ Hospital Kasur. Informed verbal and written consent was obtained from all participants.

## Results

A total of 100 patients were included in this audit. The study population had a mean age of 42.4 ± 8.2 years (range: 18-55 years), with the majority of the cohort (72%, n = 72) falling within the 35- to 49-year age bracket. Unless otherwise specified, patient satisfaction and experiential metrics were assessed using a standardized five-point Likert scale (1 = very dissatisfied, 5 = very satisfied). Table 1 summarizes the continuous and categorical demographic variables of the cohort.

**Table 1:** Summary of continuous and categorical demographic variables (n = 100)

Variable	Statistics (mean ± SD) or n (%)	Range
Age (years)	42.4 ± 8.2	18-55
Dialysis duration (years)	2.8 ± 1.5	0.5-10
Gender (male/female)	65 (65.0)/35 (35.0%)	N/A
Employed (yes/no)	0 (0%)/100 (100%)	N/A

Demographic and clinical profile

**Figure 1:**
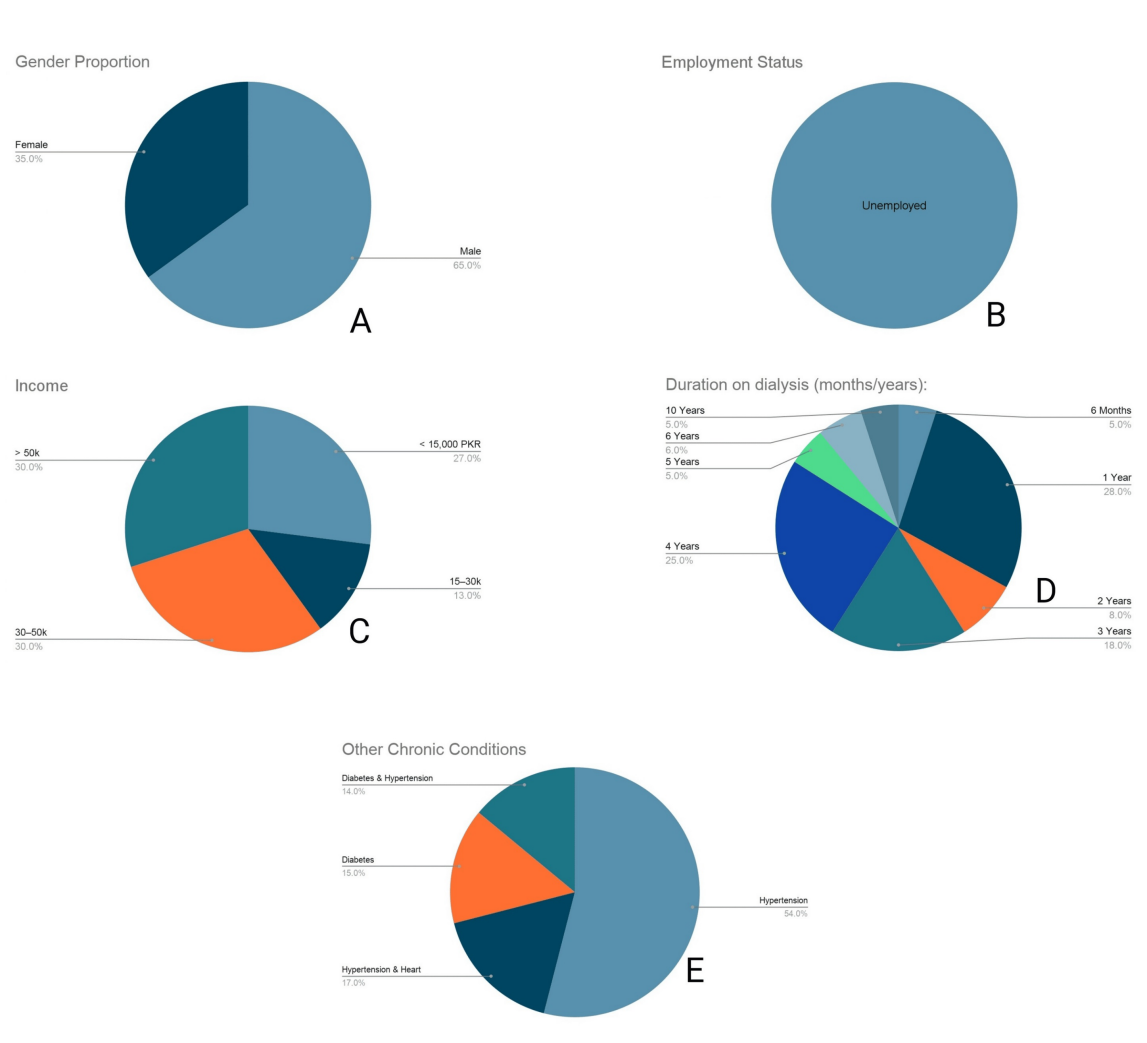
Demographic and clinical profile of the cohort (n = 100) (A) Gender distribution. (B) Employment status. (C) Monthly income in PKR. (D) Duration on dialysis. (E) Distribution of overlapping chronic comorbidities.

The cohort consisted of 65.0% (n = 65) males and 35.0% (n = 35) females (Figure 1A). Regarding employment, 100% (n = 100) of respondents reported being currently unemployed due to the demands of their treatment (Figure 1B). The 100% unemployment rate reflects a total loss of vocational productivity; this includes retirees and homemakers who reported being physically unable to engage in income-generating or household-sustaining labor due to fatigue associated with their treatment regimen.

Monthly income distribution varied: 30.0% (n = 30) reported earning 30,000-50,000 PKR, 30.0% (n = 30) reported >50,000 PKR, 13.0% (n = 13) reported 15,000-30,000 PKR, and 27.0% (n = 27) earned <15,000 PKR (Figure 1C). Dialysis treatment duration ranged from six months to 10 years, with the highest concentrations at the one-year (28.0%, n = 28) and four-year (25.0%, n = 25) milestones; 5.0% (n = 5) of participants had been on dialysis for 10 years (Figure 1D).

Comorbidities were highly prevalent and categorized into mutually exclusive groups to account for overlap. Hypertension was the most frequently reported condition overall (85.0%). When stratified, 54.0% (n = 54) had standalone hypertension, 17.0% (n = 17) had concurrent hypertension and heart disease, and 14.0% (n = 14) had concurrent hypertension and diabetes mellitus. An additional 15.0% (n = 15) presented with standalone diabetes mellitus (Figure 1E). 

Technical clinical care and safety standards

**Figure 2:**
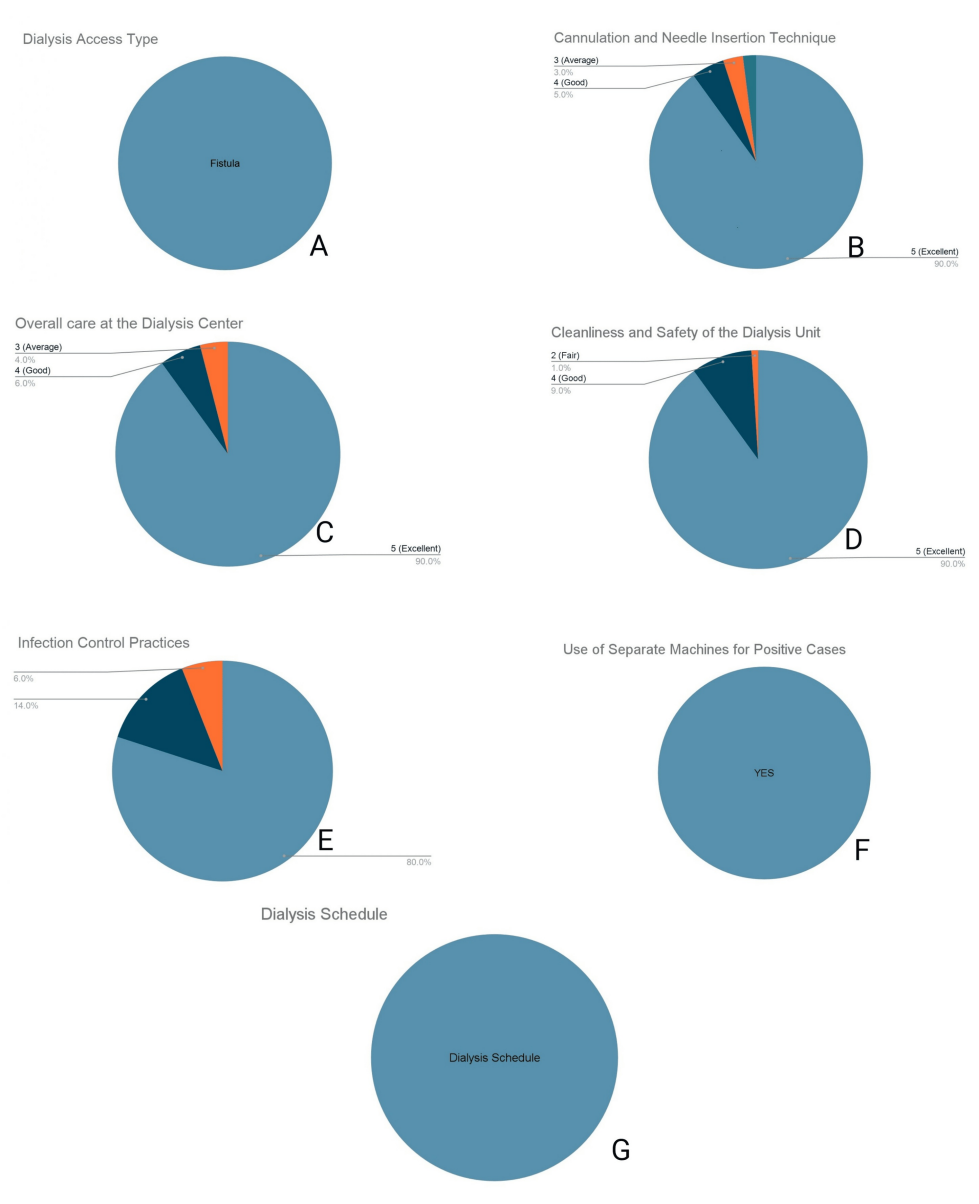
Technical clinical care and safety standards (A) Dialysis access type utilized. (B) Patient satisfaction with cannulation technique. (C) Rating of overall facility care. (D) Rating of unit cleanliness and safety. (E) Rating of infection control practices. (F) Patient confirmation of dedicated machines for positive cases. (G) Rating of schedule punctuality.

All participants (100%, n = 100) utilized an AVF as their primary dialysis access (Figure 2A). Satisfaction with technical care was high; regarding cannulation and needle insertion technique, 90.0% (n = 90) of respondents awarded a maximum score of 5, and 5.0% (n = 5) awarded a score of 4 (Figure 2B). Perceptions of overall care at the dialysis center followed a similar trend, with 90.0% (n = 90) reporting a score of 5 (Figure 2C). For unit cleanliness and safety, 90.0% (n = 90) of the cohort provided a score of 5 (Figure 2D). Safety protocols demonstrated strict adherence, with 80.0% (n = 80) of patients rating infection control practices with a score of 5 (Figure 2E), and 100% (n = 100) of respondents confirming the use of separate, dedicated machines for hepatitis B/hepatitis C-positive patients (Figure 2F). Punctuality of the dialysis schedule was rated uniformly, with 100% (n = 100) of the cohort reporting a score of 5 (Figure 2G).

Patient education and communication

**Figure 3:**
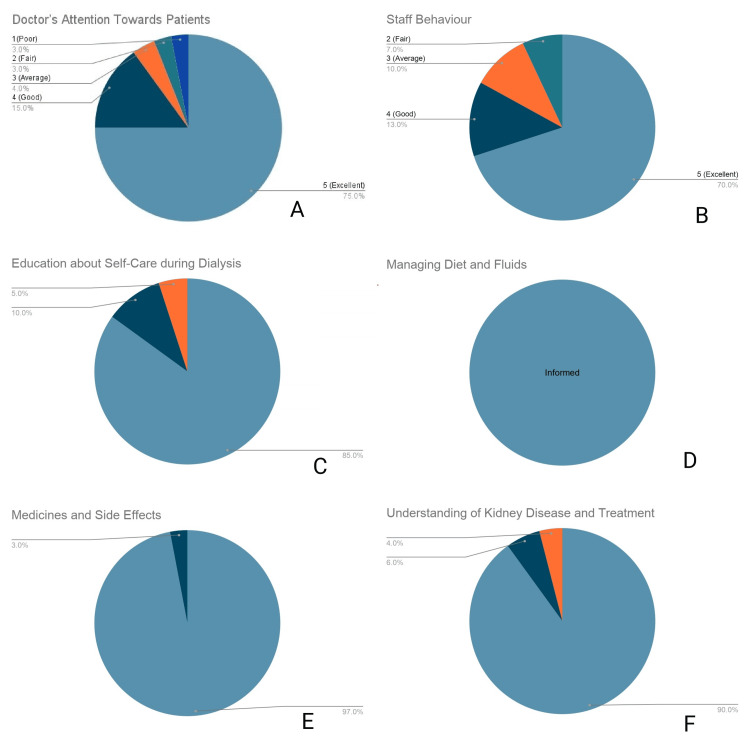
Patient education and communication (A) Satisfaction with doctor’s attention and explanation. (B) Rating of staff behavior. (C) Quality of self-care education. (D) Quality of diet and fluid management education. (E) Education on medicines and side effects. (F) Patient understanding of kidney disease and treatment.

Communication metrics indicated high health literacy engagement between staff and patients. For the physician’s attention and clinical explanations, 75.0% (n = 75) awarded a score of 5, while 15.0% (n = 15) reported a score of 4 (Figure 3A). Staff behavior received a score of 5 from 70.0% (n = 70) of respondents (Figure 3B). Patient education on self-care yielded a score of 5 from 85.0% (n = 85) of respondents (Figure 3C). Education regarding diet and fluid management received a score of 5 from 100% (n = 100) of the cohort (Figure 3D). Furthermore, 97.0% (n = 97) reported a score of 5 for education on medications and potential side effects (Figure 3E). Consequently, 90.0% (n = 90) of respondents rated their overall understanding of kidney disease and treatment with a maximum score of 5 (Figure 3F).

Psychosocial, physical, and patient experience

**Figure 4:**
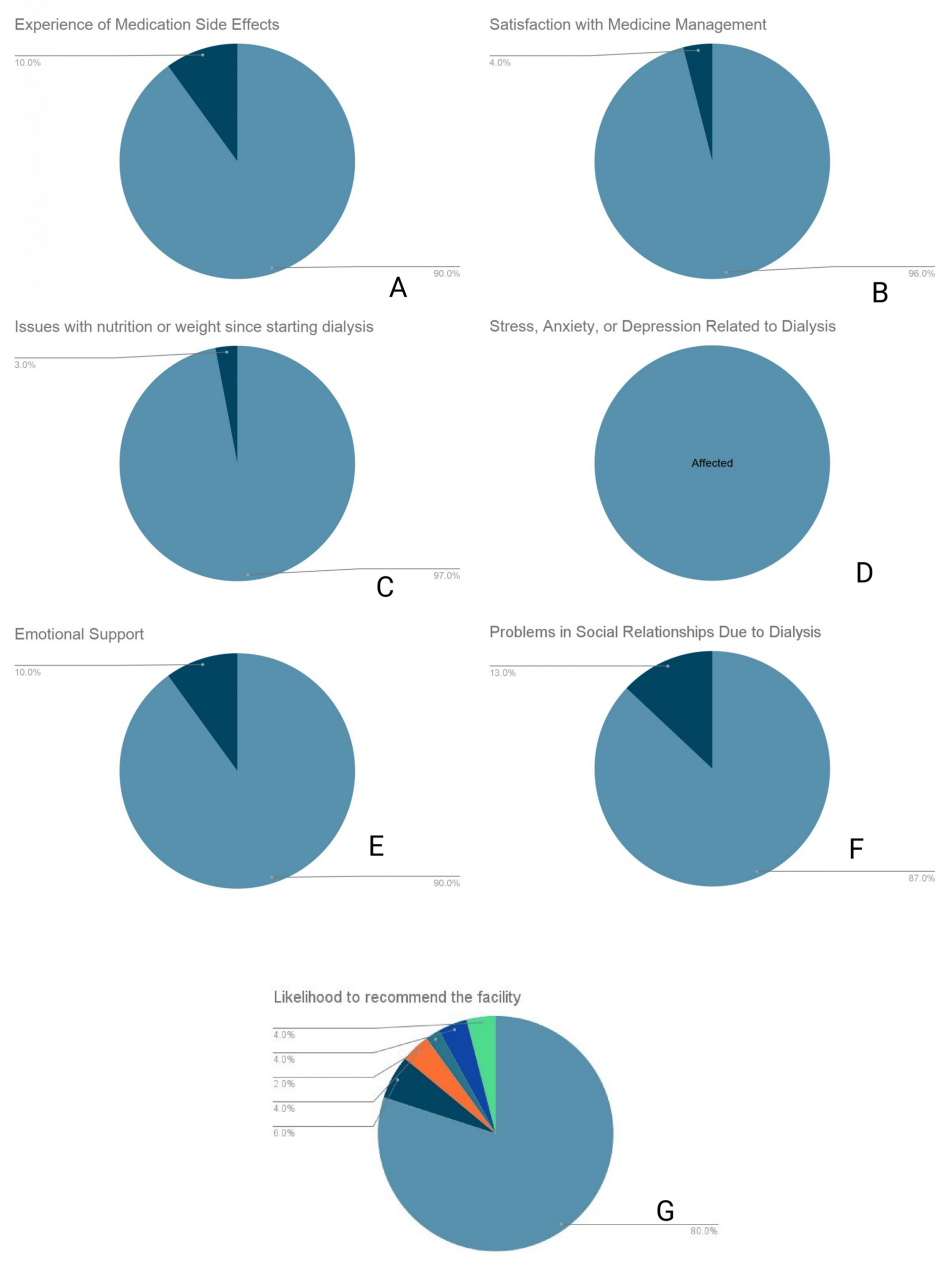
Psychosocial, physical, and patient experience outcomes (A) Prevalence of medication side effects. (B) Satisfaction with medicine management. (C) Prevalence of nutrition or weight issues. (D) Self-reported stress, anxiety, or depression. (E) Perceived emotional support. (F) Impact on social relationships. (G) Likelihood to recommend the facility (10-point scale).

Despite high technical satisfaction, the physical and psychosocial burden was notably prevalent. Treatment-related complications were frequently reported, with 90.0% (n = 90) experiencing medication side effects (Figure 4A). However, satisfaction with clinical medicine management remained high, with 96.0% (n = 96) awarding a score of 5 (Figure 4B). Physically, 97.0% (n = 97) reported issues related to nutrition or weight changes since commencing dialysis (Figure 4C). Psychologically, 100% (n = 100) of respondents reported a score of 5 (indicating severe distress) regarding treatment-related stress, anxiety, or depression (Figure 4D). Conversely, perceived emotional support remained high, with 90.0% (n = 90) awarding a score of 5 for support received (Figure 4E), and 87.0% (n = 87) reporting a score of 5 regarding the management of social relationships during treatment (Figure 4F). When asked to rate their likelihood to recommend the dialysis center on an extended 10-point scale, 80.0% (n = 80) of participants awarded a maximum score of 10, and 6.0% (n = 6) provided a score of 9 (Figure 4G).

## Discussion

This clinical audit provides a detailed snapshot of patient satisfaction, clinical practices, and the psychosocial burden among 100 hemodialysis participants in semi-urban Punjab. Overall satisfaction with technical clinical care was high, with 90% of respondents awarding the maximum score for cannulation technique and 100% reporting that their prescribed medicines were explained clearly. However, the psychological and economic findings reveal a more complex reality: 100% of participants reported that dialysis has negatively affected their work or income, and 100% reported experiencing stress, anxiety, or depression related to their treatment. Taken together, these results demonstrate a common pattern seen in South Asian renal care: high clinical and technical proficiency in the dialysis unit, accompanied by a substantial socioeconomic and mental health burden on the patient outside of the clinical procedure [8].

Comparison with local and international studies

When comprehensively contextualized against global nephrology benchmarks, this study reveals a dichotomy: high patient-perceived technical proficiency and adherence to reported safety protocols contrast with severe, unmitigated socioeconomic and psychosocial patient vulnerabilities. Clinically, the audited facilities demonstrated high adherence to isolation protocols for hepatitis-positive patients (100%) and widespread utilization of AVFs. This aligns with the rigorous standards mandated by global KDIGO and KDOQI guidelines [5,11] and is consistent with the technical benchmarks documented in large-scale Dialysis Outcomes and Practice Patterns Study audits across high-income nations [12,13].

However, the operational realities of these resource-constrained settings, characterized by high patient volumes and limited machinery, result in systemic deviations from the internationally recommended thrice-weekly dialysis schedule [2], potentially placing this cohort alongside other LMICs that may face infrastructure-driven challenges, suggesting the possibility of a local “dose-adequacy gap” that requires further objective study [14]. Furthermore, while the audit identified clear physician-patient communication regarding treatment regimens, a “knowledge-practice gap” remains. Theoretical health literacy frequently fails to translate into practical dietary compliance due to cultural habits, financial limitations, and the physiological complexities of the disease [3,15].

Addressing the satisfaction paradox

Notably, the universal (100%) prevalence of treatment-related anxiety and the reported economic vulnerability among these patients differ from the 30-45% psychological burden observed in Western audits, where integrated psychiatric and social work support is standard [16,17]. The observed convergence of high technical satisfaction and universal psychological distress in this audit may be partially attributed to “courtesy bias,” where patients undergoing life-sustaining therapy express high satisfaction with staff as a form of gratitude, despite their internal emotional suffering. Alternatively, it suggests that technical clinical excellence is independent of, and insufficient to mitigate, the financial and emotional challenges common in South Asian settings. This echoes broader literature, which identifies out-of-pocket healthcare expenditures as a primary barrier to survival and holistic rehabilitation [4,7]. Ultimately, these findings suggest that while localized secondary care centers successfully meet critical biomedical safety benchmarks, the current clinical paradigm requires the integration of robust mental health and socioeconomic support systems to meaningfully address the comprehensive burden of ESRD [9,18].

Key pattern explanations from our data

The patterns identified in this study reveal a “clinical-psychological disconnect.” High satisfaction with technical care and safety protocols, such as 100% adherence to infection control, demonstrates that while the facilities successfully meet biomedical benchmarks, these external service successes do not mitigate the intrinsic, patient-centered psychological distress associated with chronic illness. This distress exists as an internal entity separate from clinical satisfaction, further exacerbated by acute economic vulnerability; 100% of the cohort experienced a loss of vocational productivity and income. Ultimately, these patterns underscore that because psychological and socioeconomic burdens are patient-centered rather than service-dependent, there is a critical need for an integrated care model that proactively addresses the emotional toll of the disease alongside clinical procedures [1,17].

Implications for public health policy and practice

The universal prevalence of treatment-related psychological distress and socioeconomic burden identified in this study highlights a need for public health policies in Pakistan to transition from purely clinical models to integrated, patient-centered care. Specifically, these findings suggest that health authorities should consider exploring the feasibility of embedding routine mental health screening and counseling services within dialysis units at THQ and DHQ hospitals to address the high rates of anxiety and depression among patients. Furthermore, the findings underscore the necessity for enhanced financial support systems or vocational assistance programs to mitigate the impact of dialysis on patient income, alongside continued investment in standardized patient education to improve long-term adherence to complex dietary and medicinal regimens in resource-limited settings.

Strengths and limitations

This study evaluates hemodialysis services in semi-urban Punjab by benchmarking local practices at secondary care facilities (THQ and DHQ hospitals) against international KDIGO and KDOQI standards. A primary strength of this research is the direct evaluation of secondary care performance, an under-researched setting often overlooked in favor of tertiary centers, against high-level international metrics. Furthermore, the use of an interviewer-administered format ensured a 100% completion rate and allowed for the inclusion of patients with varying literacy levels, while the detailed disclosure of our questionnaire ensures methodological transparency.

However, several critical limitations must be acknowledged. First, the study utilized a non-probability convenience sampling method at two secondary care centers in the Kasur district. Consequently, while the n = 100 sample provides a representative snapshot of these specific facilities' workload and patient experience, the results may not be fully generalizable to the broader or more diverse patient populations across all regions of Pakistan.

Second, the reliance on self-reported data introduces potential response bias. To minimize this, interviews were conducted by researchers unaffiliated with the direct clinical care team in private settings to encourage candid feedback. Despite these measures, the universal maximum scores observed likely reflect a “ceiling effect” and significant “courtesy bias” (social desirability bias) inherent in patients receiving life-sustaining therapy.

Third, the use of a custom-designed screening survey rather than psychometrically validated instruments (such as the Patient Health Questionnaire-9 or General Anxiety Disorder-7) is a significant weakness; the reported psychosocial distress represents generalized perceived patient burden rather than formal clinical psychiatric diagnoses. These results suggest that the single-item metrics used lacked the discriminatory power to distinguish between varying degrees of patient experience. The universal maximum scores should therefore be interpreted as a qualitative indicator of high perceived burden and institutional gratitude rather than an epidemiologically precise measurement. Finally, as a cross-sectional study, this provides a snapshot in time and cannot establish a causal relationship between clinical practices and long-term outcomes.

## Conclusions

Conclusively, this clinical audit delineates a profound dichotomy within the secondary care hemodialysis services of semi-urban Punjab, Pakistan. While facilities such as THQ Chunian and DHQ Kasur demonstrate remarkable technical proficiency, evidenced by stringent adherence to critical infection control protocols and international vascular access standards, these clinical triumphs are fundamentally eclipsed by the unmitigated psychosocial and economic vulnerabilities of the patient population. The universal prevalence of treatment-related anxiety, depression, and catastrophic vocational loss underscores the inherent inadequacy of a purely biomedical approach to ESRD in resource-limited settings. To bridge the gap between current localized practices and holistic international benchmarks, health policymakers and administrators must urgently evolve the standard of care to integrate routine psychiatric intervention, specialized nutritional counseling, and robust socioeconomic assistance programs. Ultimately, optimizing long-term patient outcomes necessitates a paradigm shift from treating failing kidneys in isolation to addressing the comprehensive well-being of the individual.

## Appendices

Appendix A: Dialysis Patient Satisfaction Survey

This questionnaire aims to assess patient satisfaction and quality of dialysis care. The information collected will be used for research purposes only and will remain confidential.

Co-principal investigators: Dr. M. Arslan Shahid, Dr. Zeersak Laila, M. Zeeshan Arif, and Yousuf Shahjahan

1. A. Demographics - Age (in years): ________________________

2. Gender: (Mark only one oval) ( ) Male ( ) Female ( ) Other

3. Marital status: (Mark only one oval) ( ) Single ( ) Married ( ) Widowed ( ) Divorced

4. Employment status: (Mark only one oval) ( ) Employed ( ) Unemployed ( ) Retired ( ) Student

5. Duration on dialysis (months/years): ________________________

6. Dialysis frequency: (Mark only one oval) ( ) Once/week ( ) Twice/week ( ) Thrice/week

7. Do you use a separate dialysis machine for hepatitis B or C? (Mark only one oval) ( ) Yes ( ) No

8. If yes, which type of hepatitis machine do you use? (Mark only one oval) ( ) Hepatitis B ( ) Hepatitis C ( ) Both

9. Monthly income: (Mark only one oval) ( ) <15,000 PKR ( ) 15-30k ( ) 30-50k ( ) >50k

10. Dialysis access type: (Mark only one oval) ( ) Fistula ( ) Graft ( ) Catheter ( ) Other

11. Other chronic diseases: (Tick all that apply) ( ) Diabetes ( ) Hypertension ( ) Heart Disease ( ) Other

12. Overall care at the dialysis center: (Mark only one oval) ( ) 1 ( ) 2 ( ) 3 ( ) 4 ( ) 5

13. Staff behavior and communication: (Mark only one oval) ( ) 1 ( ) 2 ( ) 3 ( ) 4 ( ) 5

14. Doctor's attention and explanation: (Mark only one oval) ( ) 1 ( ) 2 ( ) 3 ( ) 4 ( ) 5

15. Cannulation/needle insertion technique: (Mark only one oval) ( ) 1 ( ) 2 ( ) 3 ( ) 4 ( ) 5

16. Cleanliness and safety of the dialysis unit: (Mark only one oval) ( ) 1 ( ) 2 ( ) 3 ( ) 4 ( ) 5

17. Dialysis performed on time / scheduled regularly: (Mark only one oval) ( ) 1 ( ) 2 ( ) 3 ( ) 4 ( ) 5

18. Infection control practices: (Mark only one oval) ( ) 1 ( ) 2 ( ) 3 ( ) 4 ( ) 5

19. Quality of education about self-care during dialysis: (Mark only one oval) ( ) 1 ( ) 2 ( ) 3 ( ) 4 ( ) 5

20. Quality of education about managing diet/fluids: (Mark only one oval) ( ) 1 ( ) 2 ( ) 3 ( ) 4 ( ) 5

21. Quality of education about medicines and side effects: (Mark only one oval) ( ) 1 ( ) 2 ( ) 3 ( ) 4 ( ) 5

22. Patient's understanding of kidney disease and treatment: (Mark only one oval) ( ) 1 ( ) 2 ( ) 3 ( ) 4 ( ) 5

23. D. Mental health and quality of life - Has dialysis affected your work or income? (Mark only one oval) ( ) Yes ( ) No

24. Emotional support from family/friends/staff: (Mark only one oval) ( ) 1 ( ) 2 ( ) 3 ( ) 4 ( ) 5

25. Stress, anxiety, or depression related to dialysis: (Mark only one oval) ( ) 1 ( ) 2 ( ) 3 ( ) 4 ( ) 5

26. Problems in social relationships due to dialysis? (Mark only one oval) ( ) Yes ( ) No

27. Prescribed medicines explained clearly: (Mark only one oval) ( ) 1 ( ) 2 ( ) 3 ( ) 4 ( ) 5

28. Experienced side effects from medicines? (Mark only one oval) ( ) Yes ( ) No

29. Satisfaction with medicine management: (Mark only one oval) ( ) 1 ( ) 2 ( ) 3 ( ) 4 ( ) 5

30. Received advice on what to eat and avoid? (Mark only one oval) ( ) Yes ( ) No

31. Follow dietary recommendations: (Mark only one oval) ( ) 1 ( ) 2 ( ) 3 ( ) 4 ( ) 5

32. Issues with nutrition or weight since dialysis? (Mark only one oval) ( ) Yes ( ) No

33. Aware of dedicated machines for hepatitis B or C? (Mark only one oval) ( ) Yes ( ) No

34. If hepatitis B/C positive, assigned a separate machine? (Mark only one oval) ( ) Yes ( ) No ( ) Not applicable

35. H. Additional comments - Number of missed dialysis sessions in the last three months: ________________________

36. On a scale of 0-10, how likely are you to recommend this dialysis center to others? (Mark only one oval) ( ) 0 ( ) 1 ( ) 2 ( ) 3 ( ) 4 ( ) 5 ( ) 6 ( ) 7 ( ) 8 ( ) 9 ( ) 10
